# Deep learning assisted analysis of biomarker changes in refractory neovascular AMD after switch to faricimab

**DOI:** 10.1186/s40942-025-00669-2

**Published:** 2025-04-11

**Authors:** Michael Hafner, Franziska Eckardt, Jakob Siedlecki, Benedikt Schworm, Tina R. Herold, Ben Asani, Siegfried G. Priglinger, Johannes B. Schiefelbein

**Affiliations:** Department of Ophthalmology, LMU University Hospital, LMU Munich, Mathildenstraße 8, 80336 Munich, Germany

**Keywords:** Neovascular age-related macular degeneration, Deep learning, Optical coherence tomography, Faricimab, Artificial intelligence, Chorioretinal biomarkers, Intravitreal therapy, Treatment-resistant AMD, Choroidal volume, Automated image analysis.

## Abstract

**Background:**

Artificial intelligence (AI)-driven biomarker segmentation offers an objective and reproducible approach for quantifying key anatomical features in neovascular age-related macular degeneration (nAMD) using optical coherence tomography (OCT). Currently, Faricimab, a novel bispecific inhibitor of vascular endothelial growth factor (VEGF) and angiopoietin-2 (Ang-2), offers new potential in the management of nAMD, particularly in treatment-resistant cases. This study utilizes an advanced deep learning-based segmentation algorithm to analyze OCT biomarkers and evaluate the efficacy and durability of Faricimab over nine months in patients with therapy-refractory nAMD.

**Methods:**

This retrospective real-world study analyzed patients with treatment-resistant nAMD who switched to Faricimab following inadequate responses to ranibizumab or aflibercept. Automated segmentation of key OCT biomarkers - including fibrovascular pigment epithelium detachment (fvPED), intraretinal fluid (IRF), subretinal fluid (SRF), subretinal hyperreflective material (SHRM), choroidal volume, and central retinal thickness (CRT) - was conducted using a deep learning algorithm based on a convolutional neural network.

**Results:**

A total of 46 eyes from 41 patients completed the nine-month follow-up. Significant reductions in SRF, fvPED, and choroidal volume were observed from baseline (mo0) to three months (mo3) and sustained at nine months (mo9). CRT decreased significantly from 342.7 (interquartile range (iqr): 117.1) µm at mo0 to 296.6 (iqr: 84.3) µm at mo3 and 310.2 (iqr: 93.6) µm at mo9. The deep learning model provided precise quantification of biomarkers, enabling reliable tracking of disease progression. The median injection interval extended from 35 (iqr: 15) days at mo0 to 56 (iqr: 20) days at mo9, representing a 60% increase. Visual acuity remained stable throughout the study. Correlation analysis revealed that higher baseline CRT and fvPED volumes were associated with greater best-corrected visual acuity (BCVA) improvements and longer treatment intervals.

**Conclusions:**

This study highlights the potential of AI-driven biomarker segmentation as a precise and scalable tool for monitoring disease progression in treatment-resistant nAMD. By enabling objective and reproducible analysis of OCT biomarkers, deep learning algorithms provide critical insights into treatment response. Faricimab demonstrated significant and sustained anatomical improvements, allowing for extended treatment intervals while maintaining disease stability. Future research should focus on refining AI models to improve predictive accuracy and assessing long-term outcomes to further optimize disease management.

**Trial registration:**

Ethics approval was obtained from the Institutional Review Board of LMU Munich (study ID: 20–0382). This study was conducted in accordance with the Declaration of Helsinki.

## Background

Age-related macular degeneration (AMD) has emerged as the leading cause of blindness in developed nations [1]. Vascular endothelial growth factor (VEGF) has been identified as a key factor in the development of neovascular AMD (nAMD). Intravitreal administration of anti-VEGF therapies inhibits endothelial cell proliferation, decreases vascular permeability, and prevents the formation of choroidal and macular neovascularization (CNV/MNV) [2]. As a result, the visual prognosis for patients with nAMD has significantly improved [3].

Currently, ranibizumab (Lucentis^®^, Novartis), aflibercept (Eylea^®^, Bayer), and brolucizumab (Beovu^®^, Novartis) are Food and Drug Administration (FDA) and European Medicines Agency (EMA) approved, while bevacizumab (Avastin^®^, Roche Pharma) is used off-label [4]. The activity of MNV in nAMD is commonly indicated by an increase in different optical coherence tomography (OCT) biomarkers such as mean central retinal thickness (CRT; mean retinal thickness within the 1-mm ETDRS-circle centered on the fovea), as well as the presence of fibrovascular pigment epithelium detachment (fvPED), subretinal hyperreflective material (SHRM) and macular fluid, including intraretinal fluid (IRF) and subretinal fluid (SRF) [5–7].

In 2022, Faricimab (Vabysmo^®^, Roche/Genentech) received FDA and EMA approval as a novel bispecific inhibitor of VEGF and Angiopoietin-2 (Ang-2) for treating nAMD. Ang-2 is known to promote vascular destabilization and enhance VEGF signaling through the Tie-2 pathway, contributing significantly to the pathological neovascularization seen in nAMD [8]. By inhibiting Ang-2, Faricimab may improve vascular stability and reduce abnormal vascular remodeling [2, 9]. The safety and efficacy of Faricimab have been confirmed in various studies [10–13]. Phase 3 trials LUCERNE and TENAYA suggest that Faricimab may allow for more efficient disease management by extending the interval between injections to up to 16 weeks after an initial loading phase of four monthly doses [10].

For patients experiencing an inadequate response to treatment, such as persistent macular fluid despite monthly injections or the inability to extend treatment intervals beyond 4 to 6 weeks, Faricimab’s dual mechanism of action may offer advantages [6, 14].

To gain a deeper understanding of Faricimab’s mode of action and therapeutic progression, it is crucial to go beyond conventional quantitative measures like CRT and best-corrected visual acuity (BCVA) and also evaluate various biomarkers associated with nAMD. However, the manual analysis of these OCT biomarkers and their longitudinal changes, particularly in large patient cohorts, presents significant challenges. This process is not only time-consuming and labor-intensive but also prone to variability, as the diverse morphological features of AMD add further complexity to the interpretation of these scans [15].

Using a previously trained deep learning-based semantic segmentation algorithm, we were able to quantitatively evaluate the progression of nAMD biomarkers such as fvPED, IRF, SRF, choroidal volume and SHRM over time [16]. This approach provides a comprehensive evaluation of Faricimab’s impact on disease activity in nAMD by means of AI.

This retrospective real-world study aimed to assess the efficacy and durability of Faricimab in treatment-resistant nAMD patients over a nine-month period following the initial intravitreal administration, through a comprehensive quantitative analysis of various OCT biomarkers associated with nAMD.

## Methods

### Participants

The Smart Eye Database from the Department of Ophthalmology at LMU University Hospital Munich was screened for patients treated with Faricimab for nAMD between January 2023 and September 2024. The inclusion criteria were: (i) switch to Faricimab following prior intravitreal therapy for nAMD due to an insufficient response to ranibizumab or aflibercept, characterized by the persistence of intraretinal or subretinal fluid despite 4-weekly anti-VEGF injections, or the inability to extend treatment intervals beyond six weeks (fluid recurrence after seven weeks); (ii) completion of a Faricimab loading phase consisting of four monthly injections, followed by a treat-and-extend regimen; (iii) absence of concurrent intraocular pathologies, including uveitis and infection.

#### Ethics approval

was granted by the Institutional Review Board of the Faculty of Medicine, LMU Munich, study ID: 20–0382, and the study followed the principles of the Declaration of Helsinki of 1964 and its later amendments. Data extraction was carried out completely anonymously, with no reference to patient names or IDs. Informed consent was obtained in accordance with institutional guidelines.

### Preoperative examinations

Pre-injection examinations included assessing BCVA, measuring intraocular pressure with non-contact tonometry, and performing dilated indirect fundoscopy.

Multimodal imaging was performed, including OCT and near-infrared scanning using the Spectralis HRA + OCT system (Heidelberg Engineering) at each visit. At the time of initial diagnosis, proof of MNV was given by either performing OCT-angiography and/or fluorescein angiography. Imaging was performed with a pattern consisting of 49 equally distributed B-scans covering a 20° x 20° area, using Automatic Real-Time (ART) averaging of 12 frames per section. All B-scans were included in the AI-driven biomarker quantification.

OCT data were collected at three pivotal time points: at the initiation of intravitreal Faricimab treatment (mo0), approximately three months later corresponding to the completion of the loading phase at the day of the fourth injection (mo3), and around nine months following the initial administration (mo9). All OCT images included in our study underwent manual quality assessment before segmentation, ensuring that only high-quality scans were analyzed.

### Automated quantification of biomarkers

Biomarker segmentation was performed using a deep learning-based semantic segmentation algorithm, previously developed by Asani et al. [16]. It employs a deep convolutional neural network and has demonstrated strong performance comparable to manual segmentation by retinal experts, as measured by F1-scores. The algorithm classifies each pixel in OCT B-scans, differentiating between various biomarkers and normal retinal tissue based on the Consensus Nomenclature for Reporting Neovascular Age-Related Macular Degeneration [17].

Six key biomarkers of nAMD were quantitatively analyzed: CRT, IRF, SRF, SHRM, fvPED and choroidal volume. These biomarkers were selected due to their established prognostic significance and frequent use in clinical decision-making for nAMD. They reflect both exudative changes (IRF, SRF) and fibrovascular remodeling (SHRM, fvPED) and choroidal changes (choroidal volume).

CRT was selected as the primary anatomical parameter due to its well-established role as a surrogate marker for macular swelling and overall disease activity in nAMD [7]. A significant decrease in CRT is often indicative of therapeutic efficacy and a reduction in exudative burden. To capture different aspects of disease progression, IRF and SRF were analyzed separately. IRF, located within the neurosensory retina, is closely linked to active neovascularization and visual decline, whereas SRF, another key marker of exudation, may have a less direct impact on visual acuity [18]. SHRM was included in the analysis as it represents hyperreflective subretinal tissue commonly associated with neovascular processes and visual impairment [15]. Its reduction could reflect successful suppression of pathological neovascularization. Moreover, fvPED was quantified, as PEDs contribute to chronic exudation, and their response to therapy provides valuable insight into disease stability and progression [7]. Additionally, choroidal volume was included, as it serves as a key indicator of MNV perfusion and is closely linked to nAMD pathophysiology, with VEGF playing a crucial role in choroidal vessel regulation [19, 20].

The algorithm provides quantitative outputs based on the standardized ETDRS grid [21], with CRT measured in µm and volumetric biomarkers expressed in arbitrary voxel units to ensure precision. Using voxel-based measures avoids potential distortions associated with image compression that can arise during conversion to the metric system. By prioritizing the accurate tracking of changes over time and differences in values, this approach ensures the most reliable method for capturing and analyzing relevant data.

### Structure of the applied segmentation algorithm

The model was based on a U-Net–type deep convolutional neural network [22] with 11 convolution layers, batch normalization [23], and ReLU activation functions [24]. Transposed convolutions in the decoder stage restored spatial resolution, while optimization was performed using the Adam optimizer with a categorical cross-entropy loss function. To improve robustness, an ensemble approach was applied, averaging predictions from multiple models trained using a leave-one-out scheme.

For training, 458 manually annotated macular OCT scans were used. Inter-annotator variability was assessed on an additional 30 scans labeled by three experts. Segmentation accuracy was evaluated using the Dice-Sørensen coefficient (F1-score), measuring overlap between predictions and ground truth [16].

On an independent test set of 36 images, the model demonstrated excellent segmentation accuracy, achieving near-perfect performance for SRF (F1-score: 0.98) and strong results for other biomarkers, including fvPED (0.78) and IRF (0.76). While slightly lower scores were observed for more complex structures, overall performance remained consistent with expert annotations [16].

### Data analysis and statistics

Data management was performed using Microsoft Excel (Version 16.78.3 for Mac), while statistical analyses were conducted using GraphPad Prism (Version 10.3.1 for macOS). A significance level of *p* < 0.05 was applied throughout the analysis. The Shapiro-Wilk test revealed that the data were not normally distributed; therefore, results are presented as medians with interquartile ranges (iqr). Comparisons of biomarkers across different time points were conducted using the Friedman ANOVA test. Where applicable, pairwise comparisons were performed using the Wilcoxon matched-pairs signed-rank test. Associations between dependent and independent variables were evaluated using Spearman’s correlation coefficient (r). Given the exploratory nature of this study, we did not apply formal corrections for multiple comparisons (e.g., Bonferroni or Benjamini-Hochberg procedures) to avoid inflating Type II errors and to preserve sensitivity for detecting potential associations.

## Results

### Baseline demographics

A total of 57 eyes from 52 patients with unresponsive nAMD were switched to Faricimab during the study period. Six eyes (10.5%) were excluded from the study due to loss of follow-up at nine months. Of the remaining 51 eyes, five (8.8%) were reverted to their previous treatment regimen at the discretion of the treating physician by the nine-month mark. All patients received a complete loading phase with Faricimab (four injections) prior to the re-switch. The decision to revert treatment was based on a suboptimal therapeutic response following, as indicated by increased activity biomarkers compared to the prior therapy. Specifically, four eyes were switched back to Aflibercept, and one eye was switched back to Ranibizumab.

Consequently, 46 eyes (80.7% of the initial 57 eyes) from 41 patients remained on Faricimab at nine months and were included in the analysis.

Baseline demographics are presented in Table 1. The average age at nAMD diagnosis was 74.70 ± 7.04 years, with a gender distribution of 26 women (63.4%) and 15 men (36.6%). On average, patients had received 30.22 ± 25.51 (mean ± standard deviation) anti-VEGF injections prior to switching, including 12.26 ± 12.80 injections of ranibizumab and 19.30 ± 19.71 injections of aflibercept. The nine-month follow-up analysis was conducted 276.45 ± 19.09 days after the first intravitreal administration of Faricimab. No severe ocular complications were reported during the study period, including cases of intraocular inflammation, retinal detachment, significant increases in intraocular pressure, intraocular hemorrhages, or retinal pigment epithelium tears. In summary, an average of 7.43 ± 0.78 intravitreal Faricimab injections per patient was administered throughout the study period.

**Table 1:** Baseline demographics of patients with treatment-resistant nAMD switched to faricimab

Number of patients	41
Number of eyes	46
Mean age (years)	74.70 ± 7.04
Gender	
Male	15
Female	26
Mean prior anti-VEGF injections	
Total (n)	30.22 ± 25.51
Total RBZ	12.26 ± 12.80
Total AFL	19.30 ± 19.71
Mean injections per year	8.50 ± 3.06
Last injection	
RBZ	10
AFL	36
CNV type	
I	19
II	18
III	9
Mean Faricimab injections during study	7.43 ± 0.78

This table summarizes the baseline characteristics of 41 patients (46 eyes) included in the study. Key demographic information includes age, gender distribution, and choroidal neovascularization (CNV) type. The history of prior anti-VEGF treatment is detailed, including the number of total intravitreal injections (RBZ: ranibizumab; AFL: aflibercept), mean injections per year, and the last anti-VEGF agent administered before the switch to Faricimab. Moreover, the mean number of Faricimab injections per patient during the study period is shown

### Biomarker changes

At baseline (mo0), all eyes exhibited evidence of MNV activity, indicated by the presence of IRF, SRF, or fvPED.

Three months after switching to Faricimab (at time point mo3), there was a significant reduction in all analyzed biomarkers compared to mo0. When comparing the patient cohort at baseline (mo0) to those at time point mo3, SRF decreased from 958 (iqr: 4600) voxel to 2 (iqr: 32) voxel, IRF from 22 (iqr: 400) voxel to 5 (iqr: 32) voxel, SHRM from 41 (iqr: 219) voxel to 1 (iqr: 80) voxel, fvPED from 10,320 (iqr: 17376) voxel to 7543 (iqr: 15239) voxel, and choroid volume from 224,499 (iqr: 125858) voxel to 209,143 (iqr: 107341) voxel.

Additionally, CRT, as a surrogate marker for disease activity, showed a statistically significant reduction from 342.7 (iqr: 117.1) µm at mo0 to 296.6 (iqr: 84.3) µm at mo3.

In the long run, 9 months after switch to Faricimab (at time point mo9), a significant change compared to mo0 could be found in terms of SRF (from 958 (iqr: 4600) voxel to 7 (iqr: 220) voxel), fvPED (from 10320 (iqr: 17376) voxel to 8486 (iqr: 14615) voxel) and choroid volume (from 224499 (iqr: 125858) voxel to 216446 (iqr: 127956) voxel). CRT decreased significantly from 342.7 (iqr: 117.1) µm to 310.2 (iqr: 93.6) µm. In terms of IRF (from 22 (iqr: 400) voxel to 11 (iqr: 154) voxel) as well as SHRM (from 41 (iqr: 219) voxel to 9 (iqr: 181) voxel), no statistically significant change was found from mo0 to mo9.

Notably, despite the ability to extend treatment intervals under the treat-and-extend regimen, no significant deterioration in any of the analyzed biomarkers was observed when comparing mo3 to mo9. This underscores the durability of Faricimab’s therapeutic effects, maintaining stable OCT markers even with prolonged intervals between treatments.

**Fig. 1:**
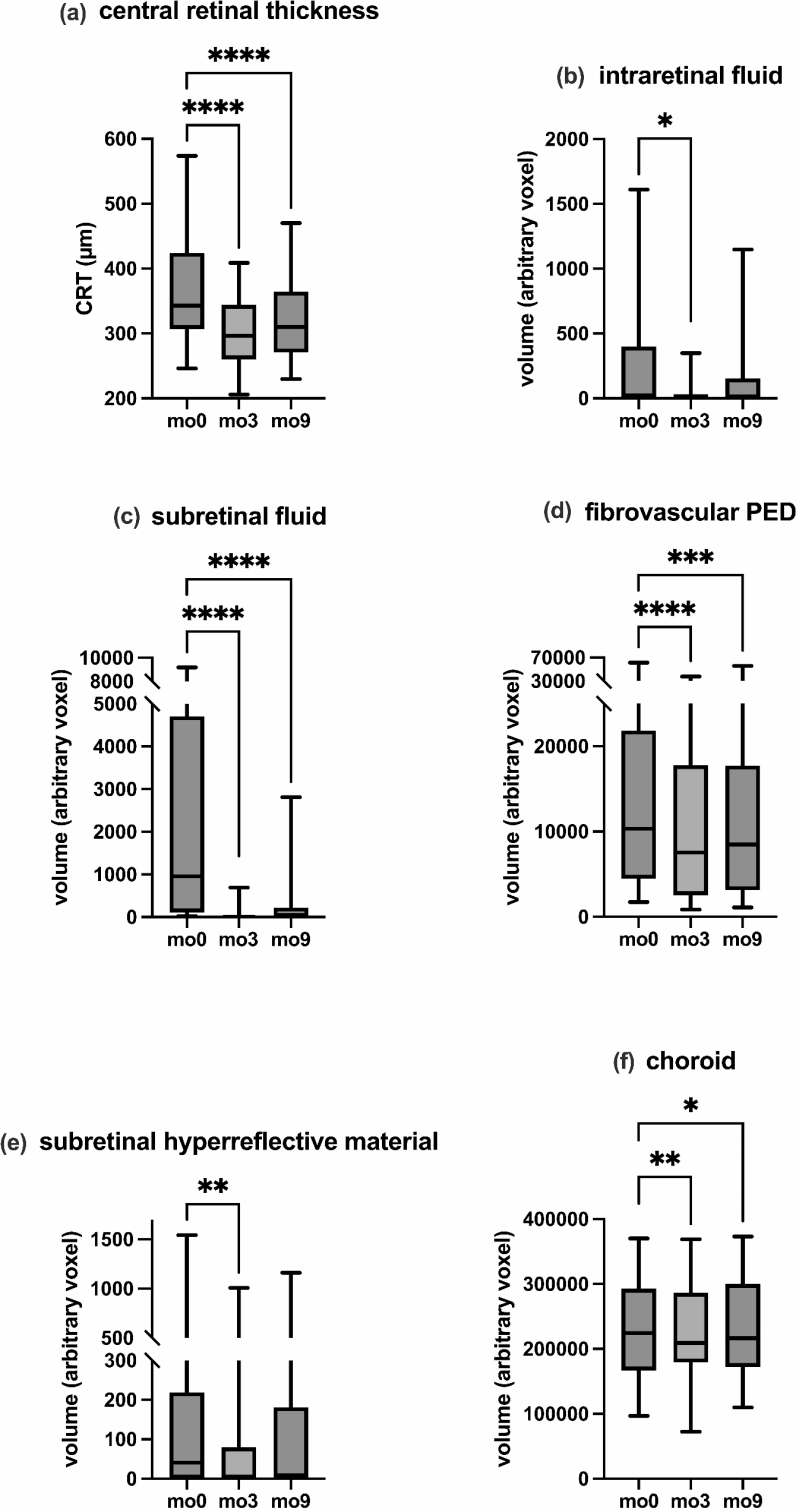
Biomarker changes following the switch to intravitreal Faricimab over a nine-month period. This figure illustrates the longitudinal changes in key OCT biomarkers after switching to intravitreal Faricimab. Biomarkers assessed include **(a)** central retinal thickness (CRT), (b) intraretinal fluid (IRF), **(c)** subretinal fluid (SRF), **(d)** fibrovascular pigment epithelium detachment (fvPED), **(e)** subretinal hyperreflective material (SHRM), and **(f)** choroidal volume. Measurements were taken at baseline (mo0, first Faricimab injection), after three months (mo3, day of fourth Faricimab injection), and nine months post-switch (mo9). Pairwise comparisons were performed using the Friedman ANOVA test, with significant *p*-values indicated by asterisks (**** *p* < 0.0001, *** *p* < 0.001, ** *p* < 0.01, * *p* < 0.05)

All data and p-values from the statistical analysis are presented in Table 2 and graphically in Fig. 1.

**Table 2:** Changes in visual acuity, biomarker levels, and treatment intervals over the study period

	BCVA (logMAR)	CRT (µm)	SRF (voxel)	IRF (voxel)	SHRM (voxel)	fvPED (voxel)	Choroid (voxel)	Injection interval (days)
mo0	0.30	342.7	958	22	41	10,320	224,499	35
mo3	0.20	296.6	2	5	1	7543	209,143	
mo9	0.20	310.2	7	11	9	8486	216,446	56
iqr (mo0)	0.40	117.1	4600	400	219	17,376	125,858	15
iqr (mo3)	0.20	84.3	32	32	80	15,239	107,341	
iqr (mo9)	0.30	93.6	220	154	181	14,615	127,956	20
*p*-value (mo0-mo3)	> 0.9999	**< 0.0001**	**< 0.0001**	**0.0399**	**0.0044**	**< 0.0001**	**0.0011**	
*p*-value (mo0-mo9)	0.7067	**< 0.0001**	**< 0.0001**	0.6475	0.1906	**0.0004**	**0.0256**	**< 0.0001**
*p*-value (mo3-mo9)	0.9815	0.0698	> 0.9999	0.6475	0.3153	0.5399	> 0.9999	

This table presents the median values (with interquartile ranges) of best-corrected visual acuity (BCVA, logMAR), central retinal thickness (CRT, µm), and key OCT biomarkers (SRF: subretinal fluid; IRF: intraretinal fluid; SHRM: subretinal hyperreflective material; fvPED: fibrovascular pigment epithelium detachment; choroidal volume) at baseline (mo0, day of switch), after three months (mo3, day of fourth Faricimab injection), and at nine months (mo9). The injection interval (days) before and after switching to Faricimab is also reported. Pairwise comparisons were conducted using the Friedman ANOVA test, with significant *p*-values (*p* < 0.05) highlighted in bold

### Treatment intervals

**Fig. 2:**
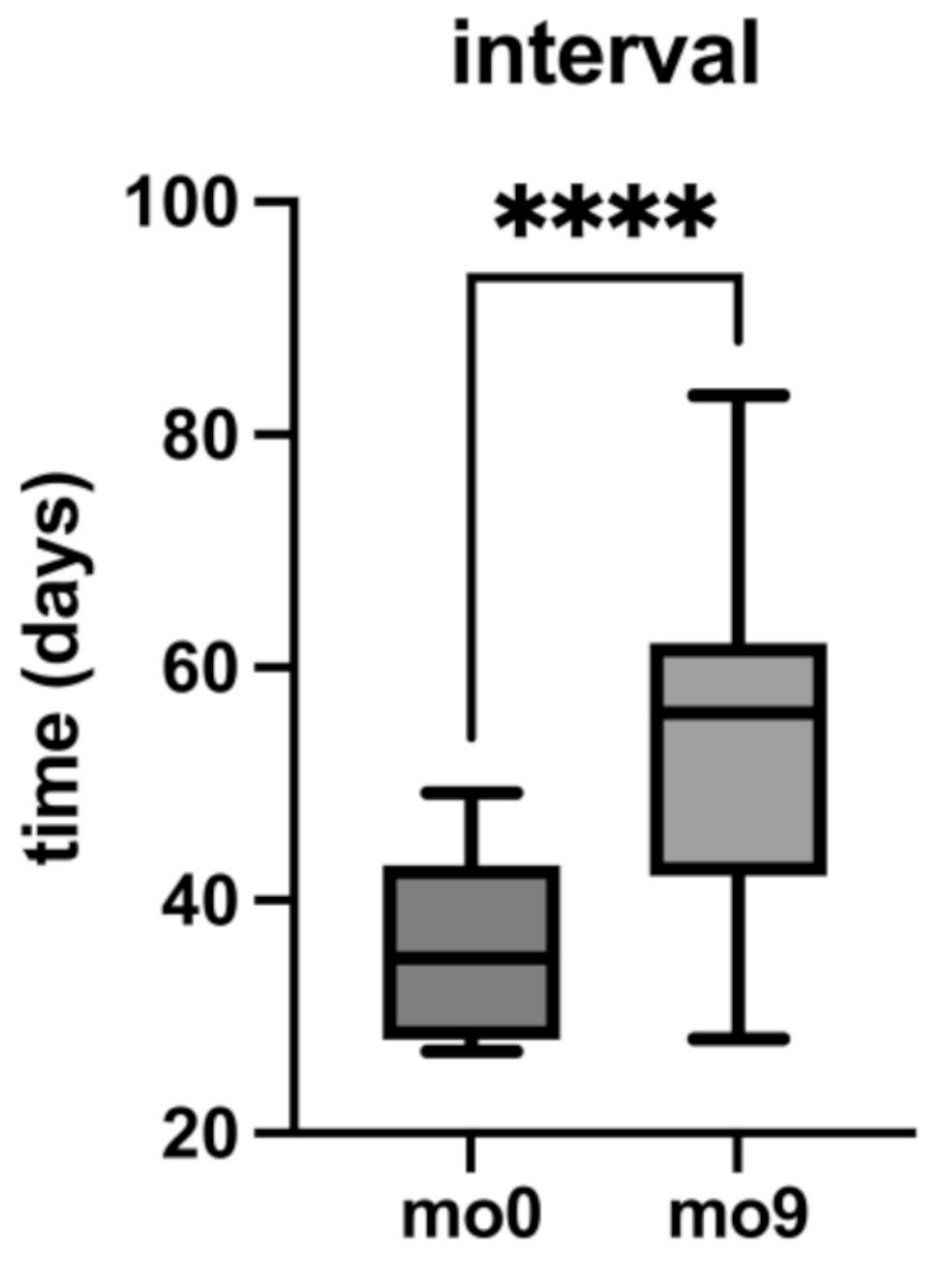
Extension of treatment intervals following the switch to intravitreal faricimab. This figure depicts the change in median injection intervals from baseline (mo0, prior to the switch) to nine months after switching to Faricimab (mo9). The treatment interval significantly increased from 35 days (interquartile range (iqr): 15) at mo0 to 56 days (iqr: 20) at mo9, representing a 60% extension. Asterisks indicate the statistical significance of pairwise comparisons (**** *p* < 0.0001)

The median injection interval was significantly prolonged from 35 (iqr: 15) days before the switch at mo0 to 56 (iqr: 20) days after the switch by the end of the follow-up period at mo9, representing a 60% extension. Results are presented in Fig. 2; Table 2.

### Visual acuity

Visual acuity (logMAR score) was preserved without any statistically significant changes throughout intravitreal treatment with Faricimab, with measurements of 0.30 (iqr: 0.40) logMAR at mo0 (before switch), 0.20 (iqr: 0.20) logMAR at mo3 and 0.20 (iqr: 0.30) logMAR at mo9. Data can be found in Table 2.

### Correlation analysis

The relationships between various biomarkers, improvements in BCVA as a key functional parameter, and the potential for extending treatment intervals were systematically analyzed. A positive correlation was identified between the change in BCVA (logMAR) from mo0 to mo9 and baseline CRT (*r* = 0.25), with a slightly weaker correlation observed for fvPED (*r* = 0.18) at mo0. This indicates that higher baseline values of these biomarkers may predict greater functional improvement in BCVA following the switch to Faricimab.

When evaluating the correlation between potential treatment interval extension (from mo0 to mo9) and baseline biomarkers, positive associations were noted with CRT (*r* = 0.25) and SRF (*r* = 0.28). A weaker correlation was also observed with fvPED (*r* = 0.17). These findings suggest that patients presenting with higher baseline CRT, greater SRF, and, to a lesser extent, larger fvPED volumes are more likely to benefit from extended treat-and-extend intervals after switching to Faricimab. A detailed summary of these findings is provided in Table 3.

**Table 3:** Correlation analysis between baseline biomarker levels and changes in BCVA and treatment interval

	ΔBCVA (mo0 - mo9)		ΔInterval (mo0 - mo9)	
	*r*	*p*-value	*r*	*p*-value
CRT (mo0)	0.25	**0.0394**	0.25	**0.0399**
IRF (mo0)	-0.06	0.6965	0.06	0.3250
SRF (mo0)	-0.01	0.9554	0.28	**0.0316**
fvPED (mo0)	0.18	**0.0422**	0.17	**0.0369**
SHRM (mo0)	-0.04	0.7831	-0.09	0.2642
Choroid (mo0)	-0.04	0.7582	-0.01	0.2236

This table displays the Spearman correlation coefficients (r) and corresponding p-values for the relationship between baseline biomarker levels and changes in best-corrected visual acuity (ΔBCVA, logMAR) and treatment interval (ΔInterval, days) from mo0 to mo9. Central retinal thickness (CRT) and fibrovascular pigment epithelium detachment (fvPED) at mo0 show a positive correlation with BCVA improvement, while CRT and subretinal fluid (SRF) levels at baseline are significantly associated with an extended treatment interval. Significant *p*-values are indicated in bold, suggesting that higher initial CRT, SRF, and fvPED values may predict more favorable treatment responses in refractory nAMD patients switched to Faricimab

## Discussion

Our findings highlight the efficacy and safety of Faricimab as a treatment for nAMD, demonstrating a significant reduction in key disease activity biomarkers. This study indicates that Faricimab not only delivers improved efficacy when transitioning from established anti-VEGF agents but also offers superior durability compared to traditional anti-VEGF monotherapies. The anatomical improvements observed on OCT scans three months after the loading phase were sustained through nine months. Furthermore, treatment intervals were successfully extended by 60% within a standard treat-and-extend regimen, even in a cohort of therapy-resistant nAMD patients characterized by severe disease activity and substantial treatment burden.

To the best of our knowledge, this is the first study to quantitatively analyze changes in single OCT biomarkers in unresponsive nAMD patients switched from ranibizumab or aflibercept in a single-center real-world setting.

While registrational trials are essential for the approval of new therapies, their strict protocols and controlled environments may not fully capture the complexities of real-world outcomes. Real-world data play a pivotal role in evaluating the effectiveness of new treatments across diverse patient populations with varying demographics, comorbidities, pre-existing conditions, and levels of treatment adherence. Such insights are critical for optimizing the practical application of new therapeutic options like Faricimab.

It could be shown that 19–27% of eyes treated with ranibizumab or aflibercept needed continuous four-week intervals [25, 26], indicating a potential benefit from therapies that offer better fluid reduction or longer intervals.

Phase 3 studies, such as TENAYA and LUCERNE, demonstrated that Faricimab achieved non-inferior outcomes compared to aflibercept with intervals of up to sixteen weeks [10]. However, these studies focused on treatment-naïve eyes, where greater improvements are expected [27].

The introduction of Faricimab as a novel treatment for nAMD has garnered considerable attention within the field of ophthalmology [27]. Our study demonstrates its high efficacy and safety profile in managing nAMD patients.

In addition to qualitatively describing the morphological changes associated with intravitreal therapy, we were able to quantify disease dynamics volumetrically by analyzing specific nAMD biomarkers in OCT scans. This was achieved using a deep learning-based semantic segmentation algorithm previously developed by Asani et al. [16]. The algorithm, trained in alignment with the Consensus Nomenclature for Reporting Neovascular Age-Related Macular Degeneration Disease Activity Biomarkers [17], enabled precise segmentation of OCT scans, facilitating robust and standardized analysis of treatment effects.

Since Faricimab’s approval, real-world trials have explored its efficacy in both short-term and long-term settings [27–37]. Many of these studies involve therapy-naïve patients with follow-up periods of three to four months. Nevertheless, the results have generally been encouraging, demonstrating either improvements or stability in anatomical features, along with maintained or improved functional outcomes. For instance, Eckardt et al. found that Faricimab can reduce central subfield thickness in therapy-recalcitrant patients who have had an insufficient response to aflibercept or ranibizumab [37]. However, long-term real-world data are still limited, partly due to the drug’s novelty.

The TRUCKEE study in the US reported significant improvements in visual acuity and CST after six months of Faricimab treatment, but observed continued improvements with additional injections, which differed from our findings. Notably, only 25% of eyes in TRUCKEE received more than one injection [35]. The study did not specify the criteria for additional injections. In contrast, in our study, the mean number of Faricimab injections received during the study period was 7.43 ± 0.78 injections per patient.

Cattaneo et al. [38] similarly investigated the efficacy of Faricimab in type 1 MNV using an AI-assisted volume quantification approach, demonstrating significant reductions in pigment epithelium detachment (PED) and fluid volumes. While their study provides valuable insights into Faricimab’s impact on PED morphology, it focused on a highly selected cohort, limited to type 1 MNV and excluding patients with type 2 and type 3 MNV. This approach limits the generalizability of their findings to broader nAMD populations. Additionally, we incorporated correlation analysis linking baseline biomarker burdens to treatment response, providing a predictive framework for individualized therapy optimization.

In general, published studies vary in their approach to the loading phase before extending treatment intervals, and the limited number of long-term studies makes comparisons challenging. Current literature supports initiating treatment with a loading phase. Preclinical approval studies suggest a 4-week interval extension for dry OCT, while our institution’s standard procedure involved a 2-week extension. Literature also supports the efficacy of a 2-week extension [12, 27, 39].

In our study, the analyzed eyes had an extensive pretreatment history, with an average of 30.22 ± 25.51 anti-VEGF injections prior to the switch. Despite the limited or inadequate response to previous therapies, transitioning to Faricimab resulted in significant improvements across nearly all evaluated nAMD OCT biomarkers, including CRT, SRF, IRF, SHRM, fvPED, and choroidal volume.

We analyzed subfoveal choroidal volume, a parameter closely linked to the pathophysiology of nAMD, as VEGF produced by the retinal pigment epithelium plays a pivotal role in regulating choroidal vessel growth [40–42]. Subfoveal choroidal volume, indicative of MNV perfusion, has been shown to influence treatment outcomes in nAMD [19]. However, existing research presents conflicting findings regarding choroidal changes during anti-VEGF therapy. VEGF inhibition may contribute to choroidal thinning through mechanisms such as vasoconstriction, reduced endothelial cell fenestrations in the choriocapillaris, or decreased CNV activity [20]. Faricimab, which uniquely targets both VEGF and the Ang1/Tie2 pathway, may exert a more pronounced effect on the underlying pathophysiology of nAMD compared to conventional anti-VEGF therapies. This aligns with our observations, where a significant reduction in choroidal volume was noted following the switch to intravitreal Faricimab, underscoring its robust influence on nAMD pathophysiology.

In line with previous studies, we observed a significant reduction in CRT over a nine-month follow-up period following the switch to Faricimab and the first intravitreal injection. Our study, however, provides a more detailed understanding of disease dynamics by quantitatively analyzing multiple OCT biomarkers. Unlike investigations with shorter follow-up durations, our analysis extends to approximately nine months within a single-center European patient cohort.

Notably, significant changes in key biomarkers (CRT, SRF, IRF, SHRM, fvPED) were predominantly observed during the loading phase with Faricimab (from mo0 to mo3), despite patients having undergone prior anti-VEGF treatment. This highlights the robust impact of the initial doses of intravitreal Faricimab in inducing morphological changes in the retina. Interestingly, minimal changes were detected beyond the loading phase, suggesting that subsequent injections primarily serve to maintain the achieved anatomical and functional stabilization.

Through correlation analysis, we identified that higher baseline CRT (as a summary parameter) and larger fvPED volumes at mo0 were positively associated with improvements in BCVA from mo0 to mo9, despite the absence of a statistically significant overall change in BCVA across the study period. These findings suggest that recalcitrant nAMD patients with substantial CRT or fvPED volumes at baseline may particularly benefit from a switch to intravitreal Faricimab, achieving not only anatomical stabilization but also potential gains in visual acuity. However, it is important to note that the positive correlations observed in this study were generally modest (*r* < 0.3).

This phenomenon can be attributed to the underlying pathophysiology and therapeutic response in recalcitrant nAMD. Patients with higher baseline CRT and larger fvPED volumes typically exhibit greater disease activity, marked by fluid accumulation and vascular instability. Faricimab, with its dual VEGF and Ang-2 inhibition [2], might have a particularly pronounced impact in these cases. The substantial reduction in fluid and resolution of pathological features observed in such patients likely accounts for their functional gains in BCVA, even though no significant overall change in visual acuity was observed across the cohort. These findings underscore Faricimab’s potential to effectively target and benefit specific subgroups within the heterogeneous nAMD population.

Additionally, we identified a positive correlation between the extension of treatment intervals (from mo0 to mo9) and the baseline levels of SRF and CRT (at mo0). A weaker correlation was observed with fvPED volume at mo0. These findings indicate that, in the context of a treat-and-extend regimen, patients with pronounced CRT, persistent SRF, and, to a lesser extent, large fvPED volumes, are more likely to achieve prolonged treatment intervals when transitioning to Faricimab therapy. It is worth noting, however, that the positive correlations identified in this study were generally modest, with correlation coefficients below 0.3. This suggests that while certain anatomical or functional parameters may be associated with treatment outcomes, the strength of these associations is relatively weak. Such modest correlations imply that these parameters alone are unlikely to serve as robust standalone biomarkers for predicting treatment response. Instead, they may contribute to a more comprehensive, multifactorial model when combined with other clinical and imaging data. The low correlation coefficients also highlight the complexity of refractory nAMD and underscore the need for larger studies to better understand the nuanced relationships between these biomarkers and therapeutic outcomes.

Although our study provides valuable insights, the nine-month follow-up period remains relatively short for a chronic disease like nAMD. Longer observation is necessary to fully assess the durability of treatment effects and determine optimal retreatment intervals. Additionally, a direct comparative analysis with other anti-VEGF agents, such as aflibercept or ranibizumab, would be crucial to better define Faricimab’s relative efficacy and its potential advantages in clinical decision-making. Future research should focus on larger, multicenter studies with extended follow-up and robust comparative analyses to establish more definitive clinical recommendations and refine personalized treatment strategies.

## Conclusion

Our study demonstrates that deep learning-based biomarker segmentation provides a precise and scalable tool for monitoring disease progression in treatment-resistant nAMD. By using AI-driven OCT analysis, we were able to quantify key anatomical changes with high accuracy, enabling objective and reproducible assessments of treatment response.

Faricimab significantly reduced CRT, SRF, IRF, and fvPED during the initial loading phase, with sustained disease stability over nine months. Importantly, treatment intervals were successfully extended by 60% without compromising anatomical or functional outcomes, underscoring its potential to reduce treatment burden in patients with refractory disease.

Correlation analysis identified baseline biomarkers– specifically CRT, SRF, and fvPED– as potential predictors of extended treatment intervals, offering valuable insights for personalized therapy optimization. Patients with high baseline CRT and large fvPED volumes also exhibited greater improvements in BCVA, suggesting that specific subgroups may derive enhanced benefits from Faricimab’s dual inhibition of VEGF and Ang-2.

Our findings support the integration of AI-assisted OCT analysis into clinical workflows to improve the precision and efficiency of disease monitoring. Future research should focus on prospective, multi-center validation studies with longer follow-up durations to further assess Faricimab’s long-term efficacy and refine predictive models for personalized treatment strategies in nAMD.

## Data Availability

The datasets generated during and/or analyzed during the current study are available from the corresponding author on reasonable request.

## Abbreviations

nAMD: neovascular age-related macular degeneration
OCT: Optical coherence tomography
IRF: Intraretinal fluid
SRF: Subretinal fluid
fvPED: fibrovascular pigment epithelium detachment
PED: Pigment epithelial detachment
CRT: Central retinal thickness
BCVA: Best-corrected visual acuity
iqr: interquartile range
VEGF: Vascular endothelial growth factor
CNV: Choroidal neovascularization
MNV: Macular neovascularization
Ang-2: Angiopoietin-2
SHRM: Subretinal hyperreflective material
